# The Effect of Vasopressin Antagonists on Maternal-Separation-Induced Ultrasonic Vocalization and Stress-Hormone Level Increase during the Early Postnatal Period

**DOI:** 10.3390/brainsci11040444

**Published:** 2021-03-30

**Authors:** Bibiána Török, Anna Fodor, Sándor Zsebők, Eszter Sipos, Dóra Zelena

**Affiliations:** 1 Institute of Experimental Medicine, 1085 Budapest, Hungary; torok.bibiana@koki.hu (B.T.); anna_@index.hu (A.F.); sipos.eszter@koki.hu (E.S.); 2 János Szentágothai School of Neurosciences, Semmelweis University, 1085 Budapest, Hungary; 3 Centre for Ecological Research, Institute of Ecology and Botany, 2163 Vácrátót, Hungary; zsebok.s@gmail.com; 4 Centre for Neuroscience, Szentágothai Research Centre, Institute of Physiology, Medical School, University of Pécs, 7622 Pécs, Hungary

**Keywords:** USV, maternal separation, pup, anxiety, vasopressin antagonists, righting reflex, negative geotaxis, ACTH, corticosterone, Brattleboro rat

## Abstract

In adults, vasopressin exerts an anxiogenic effect, but less is known about the perinatal period. As a sign of distress, rat pups emit ultrasonic vocalizations when they are separated from their mothers, known as maternal separation-induced ultrasonic vocalization (MS-USV). Previously, reduced MS-USV was reported in 7–8-day-old genetically vasopressin-deficient Brattleboro rats. Here, we aimed to examine the contributing vasopressin receptor (VR) subtypes using Wistar pups. MS-USV was recorded for 10 min, 30 min after vasopressin (V) 1aR, V1bR or V2R antagonist treatment (SR49059, SSR149415, SR121463B; 3, 10 and 30 mg/kg, intraperitoneal). Sedation was studied by the righting reflex and negative geotaxis, and finally, the stress hormone levels were measured by radioimmunoassay. The vasopressin-deficient pups showed decreased MS-USV and adrenocorticotropin levels even after a saline injection, with unchanged corticosterone levels. Thirty mg/kg of V1aR-antagonist increased the corticosterone levels. All V1bR antagonist doses decreased the MS-USV and adrenocorticotropin, while 10 + 10 mg/kg of V1aR and V1bR antagonists decreased MS-USV without influencing the stress hormones. Three mg/kg of V2R antagonist enhanced MS-USV, while 30 mg/kg increased the stress hormone levels. We confirmed that vasopressin deficiency already caused anxiolytic effects in pups. V1bRs are the most important player in connection with their adrenocorticotropin (ACTH)-regulatory role, but a combination of V1aR and V1bR antagonists might be also beneficial through other mechanisms, reducing the possibility of side effects. In contrast, antagonizing the V2Rs may be stressful due to an induction of imbalance in saltwater homeostasis.

## 1. Introduction

Arginine vasopressin (AVP) plays an important role in saltwater homeostasis and the regulation of blood pressure as a peripheral hormone [1]. Besides these functions, it also works as a neuropeptide and contributes to the regulation of learning and memory, social behavior and emotionality. Most of the AVP is synthesized in the magnocellular neurons of the hypothalamic paraventricular (PVN) and supraoptic nuclei. It may have an effect on the stress axis by strengthening the function of corticotropin-releasing hormone (CRH) in the PVN [1]. Thus, AVP can increase the CRH-induced adrenocorticotropin (ACTH) levels, then finally lead to glucocorticoid (corticosterone in rodents) secretion from the adrenal gland [1,2]. This forms the hypothalamic–pituitary–adrenocortical (HPA) axis, the fundamental component of adaptation to stress. Disturbances of these mechanisms may contribute to the development of many diseases among them being anxiety. Many research studies supported the observation that the AVP level is positively correlated with the manifestation of anxiety symptoms in adulthood [3].

The perinatal period is less studied in relation to anxiety-like behavior, most probably because of the low number of available tests. One of the best tools seems to be maternal separation-induced ultrasonic vocalization (MS-USV) [4,5]. Our previous study indicated the contribution of AVP to anxiety and the emission of MS-USV using a genetic model: the natural AVP-deficient Brattleboro strain [6]. We found significantly less anxiety-like behavior and low ACTH levels compared with the wild type, both in adults [7] as well as in pups [6]. However, the contributing receptor subtype remained to be elucidated.

AVP has three vasopressin receptors (VR) in mammals: vasopressin (V) 1aR, V1bR and V2R, with different localizations in the body [8]. V1aRs can be found in the vessels’ endothelium, and their most important function is to regulate vasoconstriction. We can also find this VR subtype in the limbic system (lateral septum, amygdala and hippocampal areas), the most important regions in the regulation of anxiety-related behaviors. V1bR signaling seems to also be important in anxiety [8]. Most of the V1bRs are present in the adenohypophysis, regulating the HPA axis. V1bR, as the main VR in the pituitary, was the main target of anxiety research in the 2000s [9]. However, among others, Bayerl et al. investigated the anxiogenic role of the VRs in the PVN of adult rats with the elevated plus maze test and found that V1aR, but not the V1bR antagonist, decreased anxiety-like behavior [10]. Moreover, in 2012, clinical studies by Griebel et al. showed that the nonpeptide V1bR antagonist SSR149415 may not be useful for the treatment of generalized anxiety disorder in adult patients [11]. V2Rs are localized in the kidney, regulating saltwater homeostasis. In the brain, its appearance is restricted to the cerebellum. Thus, its contribution to anxiety is questionable but cannot be excluded [12].

Here, we aimed to establish which VR subtype could be responsible for the observed behavioral (MS-USV) and HPA axis effects of the Brattleboro rat pups. As any stress, even a single saline injection, could influence the anxiety measured by MS-USV, we first confirmed that the effect of genetic AVP mutation was still detectable after the mild stress of a saline injection. Then, the effect of pharmacological antagonism by different VRs (V1aR-antagonist SR49059, V1bR-antagonist SSR149415 and V2R-antagonist SR121463B) was studied in increasing doses (3, 10 or 30 mg/kg) in comparison with vehicle injection in association with stress hormone changes (ACTH and corticosterone) [13]. Finally, a combination of V1aR and V1bR antagonists (10 + 10 mg/kg) was used.

## 2. Materials and Methods

### 2.1. Subjects

We used 7–8-day-old Brattleboro (*n* = 19) and Wistar (*n* = 149) male and female rat pups. There was no sex effect in either case, therefore we pulled results from two sexes together. The litter sizes of 7–10 pups, and 4–8 litters were included in each experiment, leading to 1–3 pups/treatment group/litter [14]. We chose this age group based on previous data [6]. Brattleboro rats were maintained at the Institute of Experimental Medicine in a colony started from breeder rats from Harlan, Indianapolis, IN, USA. Parental Wistar rats were purchased from Charles River (Budapest, Hungary) and were kept in the local animal facility. After birth, the litters were left undisturbed with their mothers until experimentation on postnatal day 7 or 8. The families were kept under standard laboratory conditions: a 12 h light–dark cycle with the lights on at 7:00 a.m., room temperature (20 ± 2 °C), 50–70% humidity and food and tap water ad libitum.The experiments were carried out between 10:00 a.m. and 2:00 p.m.

### 2.2. Measurements

#### 2.2.1. Maternal Separation-Induced Ultrasonic Vocalization

We measured the weights of the pups, marked them with waterproof ink and gave them an intraperitoneal (ip) injection. Afterward, they were placed back with the litter and were left undisturbed for 30 min. Then, the rat pups were separated from their mother and littermates and placed in a 2 L empty glass beaker without bedding or heating. The experiment was carried out in an empty, closed, soundproof room. MS-USV was recorded for 10 min by an ultrasonic sensitive frequency division detector (CIEL Electronique, CDB205 R2) connected to a personal computer and used Audacity 2.0.5 free software. The detector was put on a platform 10 cm to the side from the top opening of a glass beaker. The recordings were later analyzed by a rat call counter developed by S. Zsebők [6]. The signals were filtered, and the power spectrum was analyzed, ranging from 30 kHz to 50 kHz. In previous studies, the large portions of MS-USV emitted by the 8-day-old rats were found from 30 kHz to 50 kHz [15,16]. The threshold value was set at a signal amplitude of 0.4 V to exclude background noise. The MS-USV duration and number of calls were studied in each group. The MS-USV duration was the sum of the emitted USV durations in seconds during the 10 min observation period, and the number of calls was the average number of emitted USVs per minute.

#### 2.2.2. Testing Sedative Side Effects

At the end of 10 min of MS-USV detection, possible sedative side effects were evaluated.

For the righting reflex, the rat pups were placed on their back on a smooth, flat surface, and the latency necessary to reach the normal upright position with all four feet on the table was measured. The cut-off time was 15 s.

For the negative geotaxis, the offspring were placed on a 45° inclined foam rubber board with their nose pointing down. The animals had 30 s to rotate their body through 180°.

Both tests are widely used to assess neurobehavioral development and evaluated as being positive (the pup can conduct it within the given timeframe) or negative [17]. At this age, a control animal should be able to perform well, and a negative outcome could be judged as a sedative effect of the treatment.

#### 2.2.3. Hormone Measurements

Right after the sedative tests, the rats were decapitated, and trunk blood was collected into ice-cold Eppendorf tubes and centrifuged at 3000 rpm for 30 min at −4 °C. The serum was stored at −20 °C until hormone measurements were conducted. From the serum samples, the ACTH and corticosterone concentrations were measured by a specific radioimmunoassay (RIA) without previous extraction. Both antibodies were developed in our institute as described elsewhere [18,19,20]. The detection limits were 4 fmol/mL for ACTH and 2.7 pmol/mL for corticosterone. The intraassay coefficients of variation were 4.7% for ACTH and 12.3% for corticosterone. From the Brattleboro rats (Experiment 1), we also collected the hypophysis of the pups to determine the AVP content. Pituitary samples were stored in 100 μL 0.1 N HCl at −20 °C and then homogenized by ultrasound and centrifuged. Then, the AVP content was measured from the hundredfold diluted supernatant using a specific RIA. The rabbit antibodies were donated by Dr. M. Vecsernyés (University of Debrecen, Debrecen, Hungary). The limit of detection was 1 pg AVP/assay tube. The intraassay coefficient of variation was 10.7%. All the samples from a particular experiment were assayed in the same RIA.

### 2.3. Experiments

#### 2.3.1. Experiment 1: Genetic AVP Deficiency

Brattleboro rats born from heterozygous (di/+) mothers and homozygous diabetes insipidus (di/di) fathers were given ip injections with physiological saline 30 min before the test and placed back with their mothers. MS-USV was measured for 10 min, and at termination, trunk blood was collected for ACTH and corticosterone measurement, and hypophysis was collected for determination of the genotype (di/+ or di/di disposition based upon the AVP content) [17].

#### 2.3.2. Experiment 2: Pharmacological AVP-Effect Deficiency

A (2A) V1aR antagonist (SR49059), (2B) V1bR antagonist (SSR149415) or (2C) V2R antagonist (SR121463B) was suspended in 0.4% Tween 80 (1µL/g volume for every animal), then delivered intraperitoneally 30 min before MS-USV recording in three different doses: 3, 10 or 30 mg/kg (a generous gift from the Sanofi-Synthélabo company). In a further experimental series, a (2D) 10 mg/kg V1aR antagonist was mixed with a 10 mg/kg V1bR antagonist. The control treatment was the solvent in a 1 µL/g volume washed with 15 µL saline. MS-USV was measured for 10 min, and at the end, the righting reflex and negative geotaxis were evaluated within 2 min. At the termination of the experiments, trunk blood was collected for ACTH and corticosterone determination.

The specificity of these drugs was strongly confirmed by previous studies, and each of them effectively influenced their main target symptom. More precisely, SR49059 showed high affinity to V1aRs in the rat liver (Ki/inhibition constant/ = 1.6 ± 0.2 nmol/L) and human platelets, adrenals and myometrium (Ki ranging from 1.1 to 6.3 nmol/L). In vivo, SR49059 inhibited the pressor response to exogenous AVP in rats with a long duration of action [21].

SRR149415 had high affinity to V1bRs (hypophysis, human: Ki = 4.2 ± 1.1 nmol/L; rat: Ki = 3.7 ± 1.3 nmol/L). Its in vivo activity could be characterized as anxiolytic-like and stress relieving [13].

The human V2R bonds SR121463 with high affinity (Ki = 0.54 ± 0.09 nmol/L) [22]. SR121463 normalized serum Na^+^ levels, abolished hyponatremia and restored normal urine excretion, urine osmolality and renal function in a rat model of cirrhosis [23].

### 2.4. Statistical Analysis

Data were expressed as mean ± SEM and analyzed using the STATISTICA 13.0 software package (StatSoft, Inc., Tulsa, OK, USA) by analysis of variance (ANOVA) using one-way ANOVA (factor = treatment). In case of the Levene’s assumption being significant, the data were transformed logarithmically, and the analysis was conducted on the transformed data. Post hoc comparison was made by the Fisher’s Least Significant Difference method, and the results were presented on the figures. Correlations were calculated by the Pearson method. The level of statistical significance was taken as *p* < 0.05.

## 3. Results

### 3.1. Genetic AVP Deficiency

**Figure 1:**
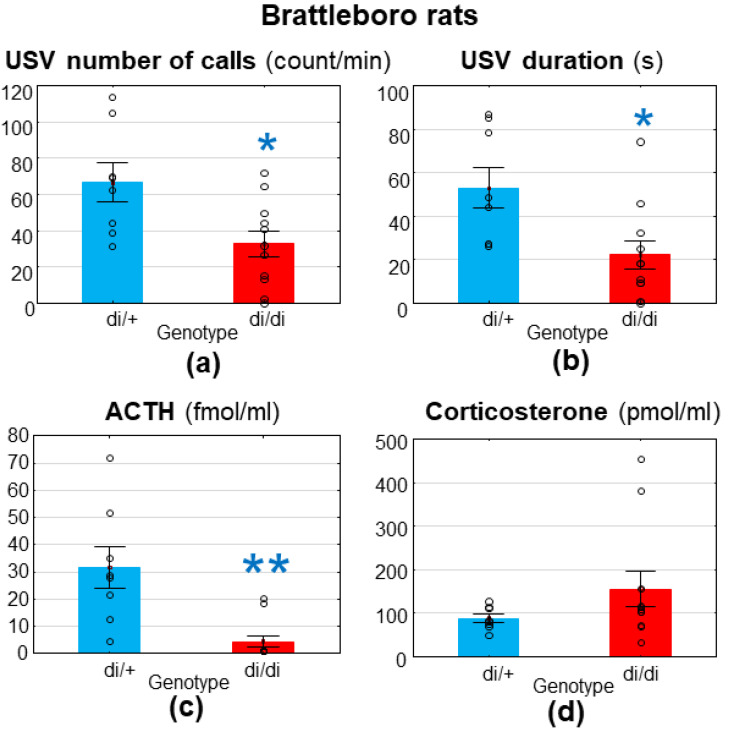
Brattleboro rats. The 7–8-day-old pups emitted reduced ultrasonic vocalization to maternal separation, measured for 10 min both in terms of (**a**) the number of calls (count/min) and (**b**) the duration (s) 30 min after a single intraperitoneal saline injection. Moreover, they showed reduced (**c**) adrenocorticotropin (ACTH, fmol/mL) elevation at the end of separation without significant changes in (**d**) corticosterone (pmol/mL) levels. di/+ = heterozygous; di/di = homozygous diabetes insipidus pups, without functional vasopressin; *n* = 8–11. * *p* < 0.05. ** *p* < 0.01 vs. di/+ control.

The AVP-deficient Brattleboro rats (di/di) were less anxious, based upon their emitted MS-USV number of calls (F_(1,17)_ = 7.600; *p* = 0.014; Figure 1a) and duration (F_(1,17)_ = 7.600; *p* = 0.014; Figure 1b) compared with their heterozygous littermates. In connection, their ACTH levels were significantly lower (F_(1,17)_ = 15.380; *p*= 0.001; Figure 1c) without significant alterations in corticosterone levels (F_(1,17)_ = 1.915; *p* = 0.184; Figure 1d).

The righting reflex (F_(1,17)_ = 1.850; *p* = 0.193) and negative geotaxis (F_(1,17)_ = 0.042; *p* = 0.840) values were comparable in the two genotypes (data not shown).

### 3.2. Pharmacological AVP Deficiency

#### 3.2.1. V1aR Antagonist

**Figure 2:**
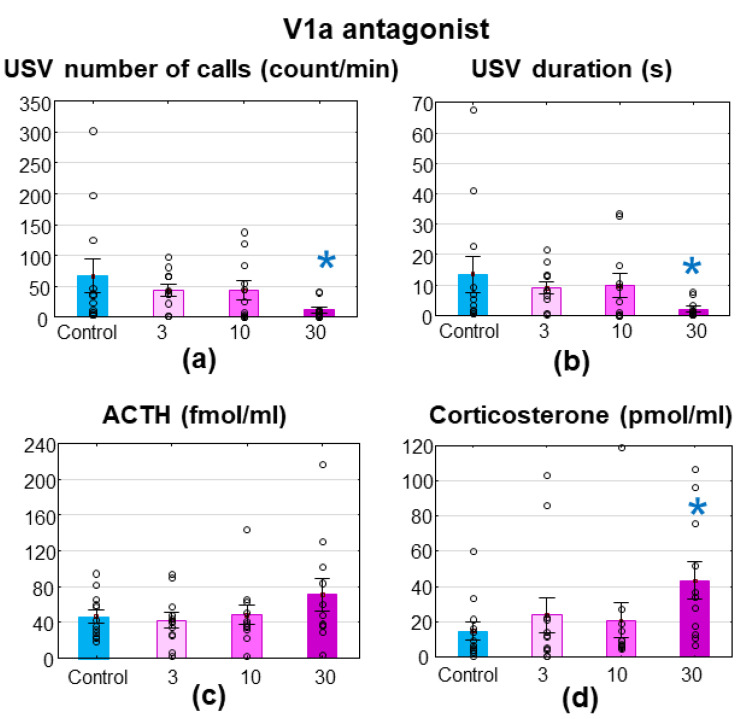
Wistar rats, treated with SR49059, a vasopressin (V) 1aR antagonist. The 7–8-day-old Wistar rat pups were treated intraperitoneally with a 3, 10 or 30 mg/kg V1aR antagonist 30 min before a 10 min maternal separation. The 30 mg/kg dose significantly reduced the emitted ultrasonic vocalization both in terms of (**a**) the number of calls and (**b**) the duration without changes in (**c**) adrenocorticotropin (ACTH, fmol/mL), but an elevation in (**d**) corticosterone (pmol/mL) levels. *n* = 12–14. * *p* < 0.05 vs. control.

The V1aR antagonist treatment decreased MS-USV only with the 30 mg/kg dose (number of calls: F_(3,42)_ = 1.788; *p* = 0.164; Fisher post hoc control vs. 30 mg/kg: *p* = 0.026; Figure 2a; duration: F_(3,42)_ = 1.551; *p* = 0.215; Fisher post hoc control vs. 30 mg/kg: *p* = 0.040; Figure 2b). The ACTH levels showed no alterations (F_(3,42)_ = 1.219; *p* = 0.315; Figure 2c), while the corticosterone levels were significantly higher in the group with the 30 mg/kg antagonist treatment (F_(3,42)_ = 1.857; *p* = 0.152; Fisher post hoc control vs. 30 mg/kg: *p* = 0.030; Figure 2d).

There was no difference between the groups in the latency of the righting reflex as well as the negative geotaxis (Table 1).

#### 3.2.2. V1bR Antagonist

**Figure 3:**
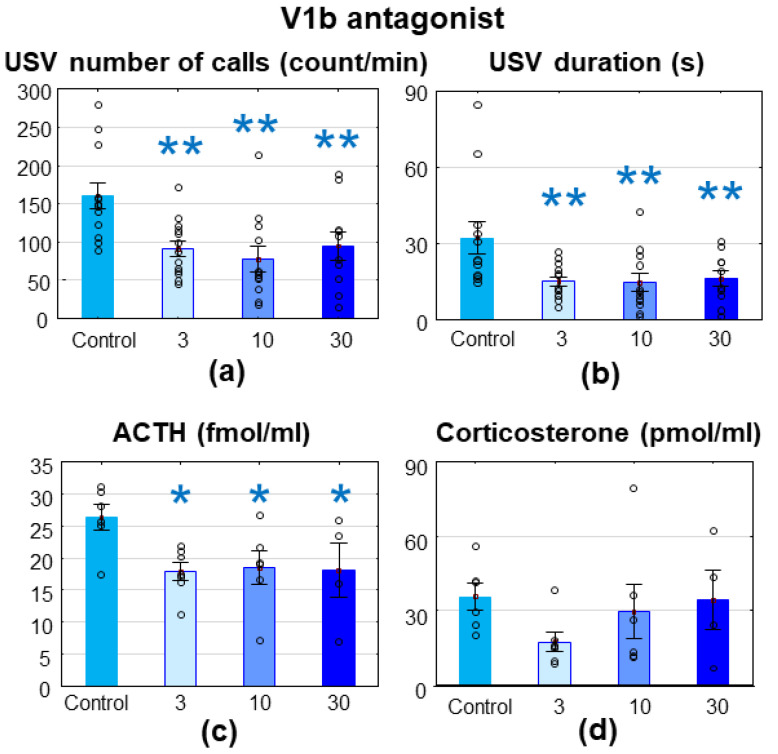
Wistar rats, treated with SSR149415, a a vasopressin (V) 1bR antagonist. The 7–8-day-old Wistar rat pups were treated intraperitoneally with 3, 10 or 30 mg/kg of the V1bR antagonist 30 min before a 10 min maternal separation. All doses significantly reduced the emitted ultrasonic vocalization both in terms of (**a**) the number of calls and (**b**) the duration and reduced (**c**) adrenocorticotropin (ACTH, fmol/mL) levels without affecting the (**d**) corticosterone (pmol/mL) values. *n* = 10–13. * *p* < 0.05. ** *p* < 0.01 vs. control.

The V1bR antagonist treatment decreased the MS-USV number of calls (F_(3,43)_ = 5.719; *p* = 0.002; Figure 3a) and duration (F_(3,43)_ = 4.470; *p* = 0.008; Figure 3b), accompanied by reduced ACTH levels (F_(3,19)_ = 3.008; *p* = 0.056; Figure 3c) without changes in corticosterone levels (F_(3,19)_ = 1.230; *p* = 0.326; Figure 3d).

There was no difference between the groups in the latency of the righting reflex and the negative geotaxis (Table 1) [24].

#### 3.2.3. V2R Antagonist

**Figure 4:**
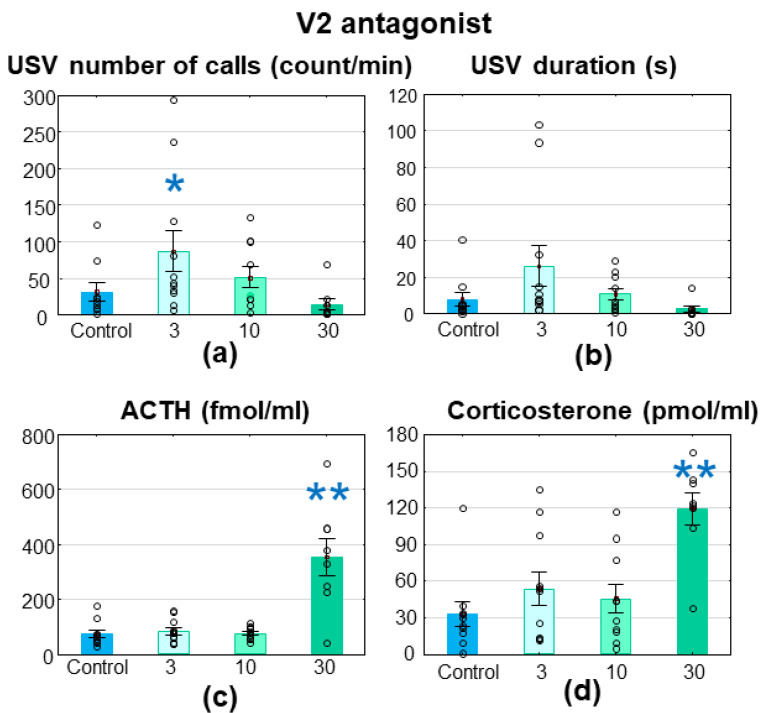
Wistar rats, treated with SR121463B, a a vasopressin (V) 2R antagonist. The 7–8-day-old Wistar rat pups were treated intraperitoneally with a 3, 10 or 30 mg/kg V2R antagonist 30 min before a 10 min maternal separation. The 3 mg/kg dose significantly enhanced the emitted ultrasonic vocalization in terms of (**a**) the number of calls, with a similar tendency in (**b**) the duration and 30 mg/kg elevated (**c**) adrenocorticotropin (ACTH, fmol/mL) and (**d**) corticosterone (pmol/mL) levels. *n* = 9–11; * *p* < 0.05, ** *p* < 0.01 vs. control.

The 3 mg/kg V2R antagonist enhanced MS-USV (number of calls: F_(3,35)_ = 4.891; *p* = 0.006; Fisher post hoc control vs. 3 mg/kg: *p* = 0.041; Figure 4a; duration: F_(3,35)_ = 4.935; *p* = 0.006; Fisher post hoc control vs. 3 mg/kg: *p* = 0.057; Figure 4b), while the higher doses had no effect on MS-USV. Both stress hormone levels were higher 45 min after a single 30 mg/kg V2R antagonist treatment compared with the control injection group (ACTH: F_(3,35)_ = 13.321; *p* = 0.000; Fisher post hoc control vs. 30 mg/kg: *p* = 0.000; Figure 4c; corticosterone: F_(3,35)_ = 8.363; *p* = 0.000; Fisher post hoc control vs. 30 mg/kg: *p* = 0.000; Figure 4d).

There was no difference between the groups in the latency of the righting reflex or the negative geotaxis (Table 1).

#### 3.2.4. V1aR + V1bR Antagonists

**Figure 5:**
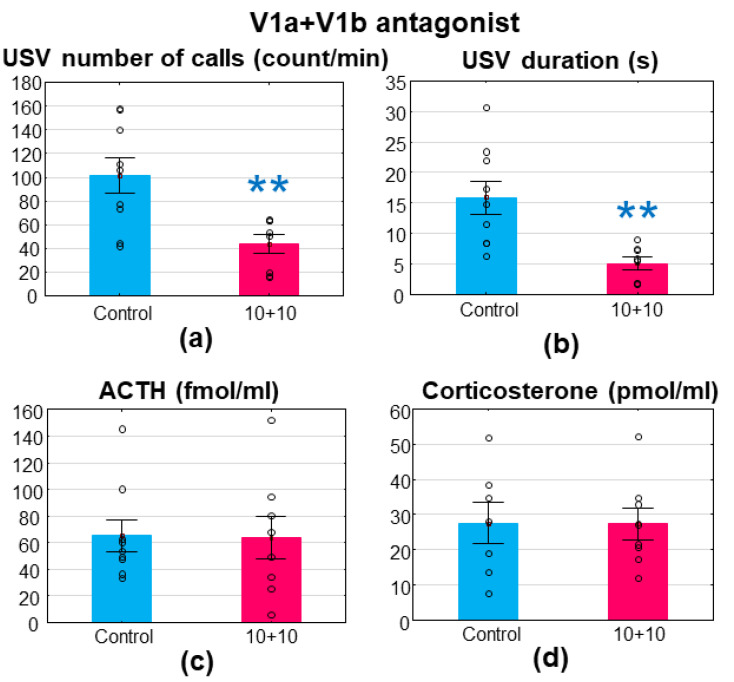
Wistar rats, treated with SR49059 (a vasopressin (V) 1aR) + SSR149415 (V1bR) antagonists. The 7–8-day-old Wistar rat pups were treated intraperitoneally with a mixture of 10 + 10 mg/kg V1aR + V1bR antagonists 30 min before a 10 min maternal separation. The combination significantly reduced the emitted ultrasonic vocalization both in terms of (**a**) the number of calls and (**b**) the duration without affecting the (**c**) adrenocorticotropin (ACTH, fmol/mL) or (**d**) corticosterone (pmol/mL) levels. *n* = 9–8. ** *p* < 0.01 vs. control.

The combination of V1aR and V1bR antagonists effectively reduced MS-USV (number of calls: F_(1,15)_ = 10.440; *p* = 0.006; Figure 5a; duration: F_(1,15)_ = 15.616; *p* = 0.001; Figure 5b) without any effect on the stress hormones (ACTH: F_(1,15)_ = 0.008; *p* = 0.931; Figure 5c; corticosterone: F_(1,15)_ = 0.001; *p* = 0.982; Figure 5d). The same dose of the V1aR antagonist induced 34.3% and 26.8% nonsignificant reductions in the MS-USV number of calls and duration, respectively, while in the case of the V1bR-antagonist, 51.5% and 54.3% significant reductions were visible. The combination induced a 57.1% reduction in the MS-USV number of calls and a 68.5% reduction in duration.

There was no difference between the groups in the latency of the righting reflex or the negative geotaxis (Table 1).

### 3.3. Correlations

**Figure 6:**
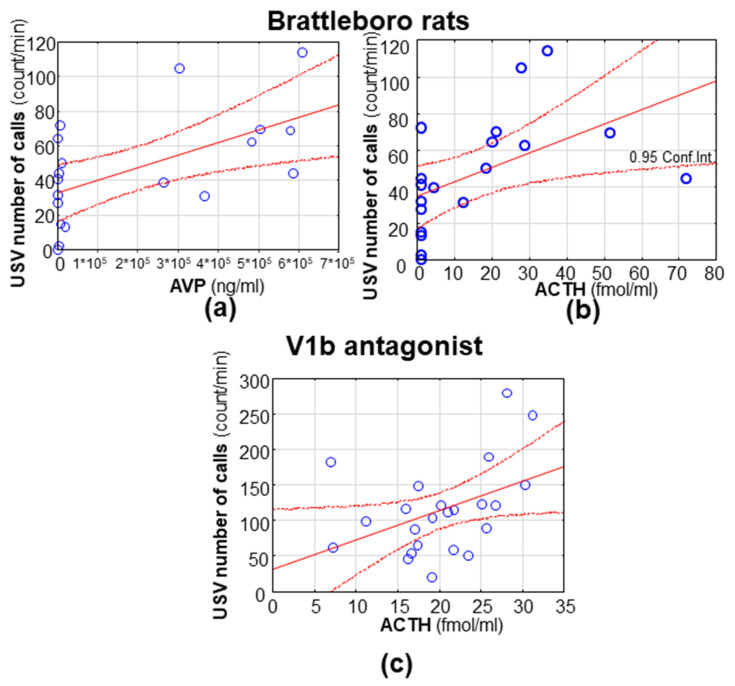
The most important correlations. In Brattleboro pups (both di/+ and di/di genotypes), (**a**) the hypophysis vasopressin (AVP) content positively correlated with the emitted number of ultrasonic vocalizations. A similar positive correlation was observable between the serum ACTH levels and the ultrasonic vocalization number both in (**b**) the Brattleboro pups and (**c**) after a vasopressin (V) 1bR antagonist treatment.

As could have been expected, the number of calls and duration of MS-USV positively correlated with each other in all experimental series. Interestingly, the same was true for ACTH and corticosterone correlation, except in the case of Brattleboro animals, where there was no correlation at all (data not shown). In the Brattleboro strain, the AVP content of the hypophysis showed a significant positive correlation with the MS-USV number of calls (r = 0.556; *p* = 0.017; Figure 6a) and duration (r = 0.541; *p* = 0.020). Moreover, in this case, the serum ACTH level also showed positive correlation with the number of emitted MS-USVs (r = 0.491; *p* = 0.038; Figure 6b). Interestingly, similar ACTH and MS-USV number of calls correlation was detected after V1bR antagonist treatment (r = 0.424; *p* = 0.044; Figure 6c).

## 4. Discussion

Our results confirmed that genetic AVP deficiency already had an anxiolytic effect during the early postnatal age, which was not influenced by the mild stress of an ip saline injection. The positive correlation between the pituitary AVP content and MS-USV further confirmed the participation of this neuropeptide in the separation-induced vocalization. Pharmacological analysis showed that a high dose (30 mg/kg) of the V1aR antagonist and all studied doses of the V1bR antagonist reduced MS-USV, while the V2R antagonist elevated it in the smallest studied dose (3 mg/kg). The number of MS-USVs correlated positively with the ACTH levels in the case of the V1bR antagonist only, similar to the Brattleboro rats. None of the studied interventions influenced the latency of the righting reflex negative geotaxis, suggesting that they were without any sedative side effects.

As anxiety is a stress-related disorder, drugs influencing the HPA axis were the focus of interest for its treatment. Indeed, the first selective and orally active nonpeptide antagonist of V1bRs (SSR149415) in adult rodent models had anxiolytic and antidepressant-like effects [25]. We also found a strong anxiolytic effect of the V1bR antagonist in rat pups with all the studied doses (3, 10 or 30 mg/kg), which confirmed our previous results with 10 [6] and 30 mg/kg [5]. In a previous experiment, the same V1bR antagonist in the same doses showed only a tendency to reduce MS-USV [26]. However, the authors used 9–11-day-old animals (for age-dependent MS-USV, see Figure 1 in [6]) and 5 min of measurement, which might be responsible for the reduced sensitivity of their assay. Similarly, the tendency seen with another V1bR-antagonist, TASP0233278 [27], might be also attributed to their less sensitive assay, as they also used 5 min of measurement and older animals (21–30 g in contrast to our 16–20 g animals). In another study on mice, the shorter recording time (5 min) might also have been responsible for the possible small difference between wild type (WT) and V1bR knockout (KO) animals [28]. However, in these V1bR KO animals, the repeated MS was not able to induce any increase in the number of MS-USV, in contrast to their WT littermates, supporting some anxiolytic role of this VR subtype.

In our hands, the positive correlation between MS-USV and stress hormones, in the case of V1bR antagonist treatment, confirmed that this VR subtype is able to influence anxiety through the regulation of stress hormones. Indeed, although SSR149415 can penetrate into the brain, its half-life is relatively short (about 60 min) [24]. Thus, it is more likely that it acts on the pituitary V1b receptors influencing the HPA axis. Interestingly, it was not the end hormone of the axis (corticosterone in rodents), but the pituitary component where ACTH was implicated in this phenomenon. This was in line with the results found in the Brattleboro rats (see Figure 6b and [5,6]), where ACTH levels did not go parallel with the corticosterone levels. Although it is hard to separate the effect of ACTH from the effect of its downstream molecules (e.g., glucocorticoids, mineralocorticoids or adrenal androgens produced in the adrenal cortex), based upon ACTH administration, many extra-adrenal effects of ACTH have been suggested (e.g., cardiovascular, metabolic, motivational or memory influencing [29]). Moreover, chronic ACTH administration in rats induced depression-like behavioral changes [30]. ACTH-producing tumors were also associated with mood swings [31]; however, in this case, the role of other factors could not be entirely excluded. Additionally, other pathways may also contribute to the V1bR-induced anxiolysis, as previous studies showed altered V1bR protein levels in the rat hypothalamus in connection with anxiolytic treatment [32].

On the other hand, several data speak in favor of the role of V1aRs in anxiety [33]. In adult rodents, both the genetic (KO mice) and pharmacological (VR antagonism) blockade of the V1aRs showed anxiolytic effects [34]. Furthermore, in adult rats, overexpression of the V1aR gene in the lateral septum increased anxiety-related behavior [35]. Our data confirmed the anxiolytic effect of V1aR antagonism in pups, but only in the highest used dose (30 mg/kg). A 10 min observation period was sufficient to reveal the anxiolytic role of another V1aR antagonist (JNJ-17308616) in 11-day-old Sprague Dawley rat pups, too [36]. Interestingly, in this study, anxiolysis was detected only in the highest (100 mg/kg) dose, when JNJ-17308616 may influence the V2Rs as well. At the periphery, V1aRs might induce vasodilatation, confirmed also by lower blood pressure in the V1aR KO mice [37]. The drop in blood pressure might be stressful; thus, it was not surprising that the highest dose of the V1aR antagonist (30 mg/kg) stimulated the HPA axis. However, this stress could hardly explain the anxiolysis. Thus, we can conclude that, in our hands, V1aR antagonists should have a central effect. Although SR49059 cannot cross the blood–brain barrier in adults [38], the increased permeability of the blood–brain barrier [39] in pups can make its anxiolytic effect possible.

The decrease in the MS-USV number and duration after combined V1aR and V1bR antagonism was higher than after any antagonist treatment alone (for exact numbers, see Section 3.2.3), without any correlation with the stress hormones. Thus, it seems that during the early postnatal period, both V1bRs and V1aRs are involved in the development of anxiety, most probably through a central brain target other than the HPA axis.

Based upon a previous finding that a V1R antagonist was able antagonize AVP administration-induced MS-USV in 8–9-day-old Sprague Dawley rat pups [40], we did not truly expect an anxiolytic effect from the V2R antagonist. In our hands, even 3 mg/kg of the V2R antagonist was anxiogenic, while 30 mg/kg was highly stressful. As V2Rs are important in saltwater homeostasis, their antagonism may induce an imbalance, leading to the appearance of anxiety and high levels of stress hormones. Once again, the HPA axis parameters were clearly separated from the MS-USV behavioral measure.

The relevance of our study is supported by the presence of AVP already in rat embryos (first appearance of its binding at embryonic day 16) [40,41,42]. At birth, the hypothalamic level of AVP is comparable to adult levels, with the same ligand selectivity and affinity. In the brains of rat pups, VR1 [42] as well as V2R subtypes [43] were found, while V2Rs were expressed at the periphery [44].

We observed interesting strain differences. First, the length of an MS-USV bout (duration/number of calls) was substantially longer in the Brattleboro (87.335 ± 5.685 ms/bout) than in the Wistar rat strain (18.995 ± 2.142 ms/bout), despite similar MS-USV frequencies (no differences were found in this parameter between the treatment groups). The Brattleboro pups (17.768 ± 0.327 g) were smaller than the age-matched Wistar rats (19.718 ± 0.242 g), suggesting possible developmental differences. However, our previous study showed that the number of MS-USV calls was higher, not lower, in a heavier pup [5]. Further, in the controls, the corticosterone values were higher in the Brattleboro than in the Wistar pups. This is consistent with our previous results [6,45]. Moreover, an earlier study found that Long Evans animals (the origin of the Brattleboro strain) were more stress reactive than the Wistar strain [46]. This different stress sensitivity of the two strains can—at least partly—explain the observed strain differences.

## 5. Conclusions

All in all, we confirmed the involvement of both V1bRs and V1aRs in the anxiolytic effect of AVP without the contribution of V2Rs. HPA axis changes can only partly contribute to the observed anxiolysis. Taking into consideration the possible side effects, a mixed V1aR/V1bR antagonist might be more beneficial than either antagonist alone.

## Figures and Tables

**Table 1:** Righting reflex and negative geotaxis values.

Antagonist Treatment	Time (s)	Doses (mg/kg)			
		0	3	10	30
V1aR	Righting	2.333 ± 0.343	2.361 ± 0.230	2.758 ± 0.397	2.212 ± 0.187
	Neg.geo.	19.861 ± 2.687	20.750 ± 2.984	18.212 ± 2.735	16.485 ± 2.929
V1bR	Righting	1.303 ± 0.156	2.279 ± 0.669	1.947 ± 0.303	2.103 ± 0.266
	Neg.geo.	9.194 ± 0.963	8.487 ± 0.880	9.528 ± 1.215	10.100 ± 1.673
V2R	Righting	2.133 ± 0.218	2.267 ± 0.325	2.033 ± 0.195	2.250 ± 0.300
	Neg.geo.	11.967 ± 1.961	13.970 ± 2.707	15.267 ± 3.010	18.667 ± 2.567
V1aR + V1bR	Righting	1.741 ± 0.282		2.750 ± 0.552	
	Neg.geo.	8.296 ± 1.301		7.125 ± 0.900	

No significant alterations were discovered. Righting = righting reflex; Neg.geo. = negative geotaxis.

## Data Availability

The data presented in this study are available on request from the corresponding author.
